# Evaluation of Early Cholecystectomy versus Delayed Cholecystectomy in the Treatment of Acute Cholecystitis

**DOI:** 10.1155/2016/4614096

**Published:** 2016-10-10

**Authors:** Miguel Sánchez-Carrasco, Juan C. Rodríguez-Sanjuán, Fernando Martín-Acebes, Francisco J. Llorca-Díaz, Manuel Gómez-Fleitas, Rocío Zambrano Muñoz, F. Javier Sánchez-Manuel

**Affiliations:** ^1^Department of General Surgery, Burgos University Hospital, Avenida Islas Baleares 3, 09006 Burgos, Spain; ^2^Department of General Surgery, University Hospital “Marqués de Valdecilla”, Avenida Valdecilla, s/n, 39008 Santander, Spain; ^3^Department of Epidemiology, Preventive Medicine and Public Health, School of Medicine, University of Cantabria, Avenida Herrera Oria, s/n, 39011 Santander, Spain

## Abstract

*Objective*. To evaluate if early cholecystectomy (EC) is the most appropriate treatment for acute cholecystitis compared to delayed cholecystectomy (DC).* Patients and Methods*. A retrospective cohort study of 1043 patients was carried out, with a group of 531 EC cases and a group of 512 DC patients. The following parameters were recorded: (1) postoperative hospital morbidity, (2) hospital mortality, (3) days of hospital stay, (4) readmissions, (5) admission to the Intensive Care Unit (ICU), (6) type of surgery, (7) operating time, and (8) reoperations. In addition, we estimated the direct cost savings of implementing an EC program.* Results*. The overall morbidity of the EC group (29.9%) was significantly lower than the DC group (38.7%). EC demonstrated significantly better results than DC in days of hospital stay (8.9 versus 15.8 days), readmission percentage (6.8% versus 21.9%), and percentage of ICU admission (2.3% versus 7.8%), which can result in reducing the direct costs. The patients who benefited most from an EC were those with a Charlson index > 3.* Conclusions*. EC is safe in patients with acute cholecystitis and could lead to a reduction in the direct costs of treatment.

## 1. Introduction

Acute cholecystitis is a pathology of inflammatory origin, usually associated with cholelithiasis, with a high incidence in our environment. The treatment of acute cholecystitis involves an important socioeconomic impact. There are two surgical therapeutic options: early cholecystectomy (EC) during the same admission or delayed cholecystectomy (DC) during a later admission after conservative treatment.

The first studies that assessed EC as a treatment for acute cholecystitis date back to the 1950s [1–3]. In 1970, the first controlled study was published by van der Linden and Sunzel, demonstrating better morbidity and shorter average hospital stay after open EC [4]. The exponential development of laparoscopic surgery occurred during the 1990s. Some of the first publications about laparoscopic EC showed bad results in terms of morbimortality and high percentages of bile duct injuries. Based on these results, laparoscopic EC was deprecated and even considered a contraindication for the treatment of acute cholecystitis, favoring initial conservative treatment followed by a laparoscopic DC. In 1998, Kiviluoto et al. reported similar results in terms of morbimortality between laparoscopic EC and open EC [5]. In that same year, Lo et al. presented the first controlled study that compared laparoscopic EC and laparoscopic DC, with lower morbidity and hospital stay in the laparoscopic EC group [6]. Recently, many studies have reported similar results in favor of laparoscopic EC. It is important to note that the vast majority of these articles only include laparoscopic cases, which could cause a bias in the external validity of these studies, as they exclude many of the less favorable cases involving open EC [7–17].

In spite of many publications that suggest benefits in favor of EC, there is still controversy regarding the timing to perform cholecystectomy. Although literature favors laparoscopic EC, most evidence comes from prospective studies specifically designed to prove this particular aspect [6–11], which probably does not reflect the worldwide clinical practice. In addition, it is well known that laparoscopic EC is not the usual practice in many hospitals [12, 17–23].

Our study aims to compare two treatment protocols for acute cholecystitis, in two similar hospital centers covering two health areas with similar characteristics and with comparable morbimortality results in laparoscopic cholecystectomy for symptomatic cholelithiasis: (A) EC, performed within 48 hours after admission, versus (B) DC, performed 2–4 months after the index episode.

## 2. Patients and Methods

### 2.1. Study Population

This is a cohort retrospective study that includes 1043 patients, consecutively treated between January 1, 2005, and December 31, 2010, for acute cholecystitis. The diagnosis was made according to the Tokyo 2013 criteria [24]. The cases of cholecystitis associated with pancreatitis, choledocholithiasis, or cholangitis and those treated with percutaneous cholecystostomy were excluded. In addition, 16 cases from EC group who were treated as DC and 10 cases from DC group who were treated as EC were also excluded due to protocol violation.

The EC group consisted of 531 patients (50.9%), treated at the University Hospital “Marqués de Valdecilla”, in Santander. This group was treated with early cholecystectomy, performed within 48 hours after admission to the Surgery Department. 473 patients (89.1%) were operated on within 72 hours of symptoms onset (<72 h) and 54 patients (10.2%) after 72 hours (>72 h). Four patients (0.7%) died before surgery, because of severe cholecystitis and comorbidity.

The DC group consisted of 512 patients (49.1%), treated at the Burgos University Hospital. This group was treated with delayed cholecystectomy, performed 2–4 months after the index episode. A total of 268 patients (52.4%) underwent elective surgery after an average of 105 days, 143 patients (27.9%) required surgery ahead of schedule (urgent surgery), and 101 patients (19.7%) received no intervention.

### 2.2. Study Variables

There are (1) postoperative hospital morbidity: overall morbidity (medical and surgical complications), surgical morbidity, and the most relevant surgical complications (bleeding, infection, and bile duct injury, according to the Strasberg classification [25]), (the severity of complications was stratified according to the Clavien-Dindo scale [26]), (2) hospital mortality, (3) days of hospital stay, (4) readmissions (note that admission for elective cholecystectomy within the DC group is not regarded as reentry), (5) admissions to the Intensive Care Unit (ICU), (6) type of surgery, (7) operating time, and (8) reoperations.

Additionally, the direct cost savings by implementation of an EC program were estimated.

### 2.3. Statistical Analysis

Frequency distributions and summary statistics were calculated for all variables; values are expressed as mean or median and range. A Kolmogorov-Smirnov test was used to study the distribution of each variable, and P-P and Q-Q charts were used to confirm it. The majority of variables did follow a normal distribution, and parametric tests were used for comparisons. The independent variable of interest was the variable EC versus DC. The endpoints of this study were the evaluation of morbidity, mortality, days of hospital stay, readmissions, admissions to ICU, type of surgery, operating time, and reoperations. Univariate analysis was performed with logistic or linear regression models or a Mantel-Haenszel chi-square test. A multivariate analysis was performed in order to analyze if the following variables were confounders between the independent and endpoints variables: age, sex, ASA degree [27], Charlson index [28], and severity of the cholecystitis. A significance level of 5% (*p* < 0.05) was accepted in all cases. SPSS software version 19.0 (SPSS, IBM Corp., Armonk, NY) was used for the statistical analysis. Additionally ROC analyses of the risk of death, the risk of complications, and the risk of reoperation are included; the area under the ROC curve shows the ability of any statistical model to predict an event. The closer it gets to 1, the greater its predictive ability will be and thus its reliability.

## 3. Results

### 3.1. Comparison of the Groups

The EC and DC groups were compared on the basis of age, sex, medical-surgical risk (ASA), Charlson index, percentage of diabetic patients, and severity of the acute cholecystitis according to the Tokyo 2013 consensus criteria [24]. The EC group had a significantly lower average age, which made the average score for the Charlson index also significantly lower in this group, due to its direct relation with age and comorbidity. Similar results were found for comorbidity (ASA) and severity of acute cholecystitis (Table 1).

### 3.2. Overall Morbidity

The morbidity in the EC group was lower than in the DC group, with statistically significant differences. Within the EC group, there were no significant differences between the morbidity of patients <72 h and the morbidity of patients >72 h. Of particular note among the DC patients was the low morbidity of patients operated on electively and the high morbidity of patients operated on urgently (Table 2).

Table 3 shows the overall postoperative complications according to the Clavien-Dindo scale, considering mild complications as grades I and II and severe complications as grades III and IV. In the multivariate analysis, we found that EC had a 39% lower risk of complications than DC (OR = 0.61; 95% CI 0.45–0.82; *p* < 0.05) and that the risk of complications increases at a rate of 17% for each point of the Charlson index (OR = 1.17; 95% CI 1.01–1.36; *p* < 0.05) and proportionally with the severity of the cholecystitis: moderate cholecystitis (OR = 2.34; 95% CI 1,70–3,23, *p* < 0.05) and severe cholecystitis (OR = 7.70; 95% CI 4.21–14.07; *p* < 0.05). The area under the ROC curve is 0.73, which indicates that the predictive ability of the model to foresee complications is not very high.

An analysis of the morbidity in terms of the Charlson index, using univariate logistic regression, was made. We observed that EC patients with Charlson index < 3 had complications in 17.6% (35/199) of cases, while DC patients with Charlson index < 3 had complications in 21.6% (32/148) of cases, (OR = 1.29; *p* > 0.05). On the other hand, EC patients with Charlson index > 3 had a 37.5% (123/328) complications rate and DC patients with Charlson index > 3 had a 47.9% (123/257) complications rate (OR = 1.51; 95% CI 1.32–1.89; *p* < 0.05).

### 3.3. Surgical Morbidity

When we analyzed only surgical complications after surgery, we observed a lower rate of complications, statistically significant, in the EC group as compared to the DC group. Within the EC group, there were no significant differences between the surgical complications of patients <72 h and the patients >72 h. In the DC group, those operated on electively did not have such low morbidity as would be expected, and of particular note is the high morbidity of the urgent surgery group (Table 2).

Table 4 shows the surgical complications according to the Clavien-Dindo scale, considering mild complications as of grades I and II and severe complications as of grades III and IV.

### 3.4. Most Relevant Surgical Complications (Table 5)

(1) *Surgical Site Infection*. The proportion of infections was significantly higher in the DC group. We found the risk of postoperative infection to be twice as high in the DC group as the EC group (OR = 1.98; 95% CI 1.78–2.17; *p* < 0.05).
(2) *Bleeding*. There were no significant differences in the frequency of postoperative bleeding.
(3) *Bile Duct Injury*. Both the bile leakage and, more specifically, major injuries of the bile duct (Strasberg D-E) had a greater tendency to occur in the DC group, but the differences were not statistically significant.

### 3.5. Mortality

In the DC group we found a higher mortality than in the EC group, but the differences were not statistically significant. Of particular note within the DC group was the low mortality of the elective surgery, the high mortality of the patients undergoing urgent surgery, and the very high mortality rate of patients who did not undergo surgery (Table 2).

It is remarkable that, in the DC group, 47 of the 92 patients (51.1%) who did not undergo surgery and did not die in hospital during the treatment died within the next three years.

The multivariate analysis showed that the risk of death was lower in the EC group (OR = 0.71), but without statistical significance (*p* > 0.05). We also found that the risk of death in both groups increased by 13% for each year of age (OR = 1.13; 95% CI 1.05–1.22; *p* < 0.05) and increased by 42% for each point in the Charlson index (OR = 1.42; 95% CI 1.01–1.98; *p* < 0.05). In relation to the severity of the cholecystitis, we found a slight increase in the risk of death in the moderate cholecystitis patients (OR = 1.52), but without statistical significance (*p* > 0.05). For its part, severe cholecystitis was associated with a high mortality (OR = 11.8; 95% CI 2.67–52.27; *p* < 0.05). The area under the ROC curve is 0.94, which confirms that the model has a very high ability to predict the risk of death.

### 3.6. Days of Hospital Stay

The average hospital stay in the EC group was significantly lower than in the DC group (Table 6).

### 3.7. Readmissions

For the analysis of hospital readmissions, we established a differentiation between admissions before cholecystectomy and readmissions after surgery. If we analyze all the cases as a whole, the EC group had a 6.8% readmission rate (*n* = 36) (all postsurgical) and the DC group a 21.9% readmissions rate (*n* = 112) (*p* < 0.05) (Table 6). Within the DC group, 18.2% of the patients required at least one readmission before surgery (*n* = 93). The percentage of readmissions after surgery in DC group was 4.6% (*n* = 19), which is not significantly lower than that of the EC group.

### 3.8. Admission to the Intensive Care Unit

We found that the percentage of EC patients that required admission to the ICU was significantly lower than the DC group. A high proportion of patients who underwent urgent surgery within the DC group required admission to the ICU (Table 6).

### 3.9. Type of Surgery

There were a greater percentage of conversions from laparoscopic to open surgery in the EC group than in the DC group (*p* < 0.05) (Table 6). On the other hand, there was no significant difference between the percentage of surgeries that were completed by laparoscopy in the EC group (58.6%) and in the DC group (61.6%).

When we matched the type of surgical approach with the severity of the acute cholecystitis, we found that in the EC group, of the 394 patients (74.7%) who underwent laparoscopic surgery, 190 patients had mild cholecystitis (48.2%), 185 patients had moderate cholecystitis (46.9%), and 19 patients had severe cholecystitis (4.8%). For their part, of the 133 patients who underwent laparotomy (25.2%), 37 had mild cholecystitis (27.8%), 76 were admitted with moderate cholecystitis (57.1%), and 20 suffered severe cholecystitis (15.0%). In the DC group, of the 279 patients (67.9%) operated on by laparoscopy, 160 had mild cholecystitis (57.3%), 116 had moderate cholecystitis (41.6%), and 3 were admitted with severe cholecystitis (1.1%). When we looked at the 132 patients who underwent laparotomy (32.1%), we found that 17 were admitted with mild cholecystitis (12.9%), 80 with moderate cholecystitis (60.6%), and 35 with severe cholecystitis (26.5%). In both EC and DC, there was a significantly higher proportion of moderate and severe cases of acute cholecystitis in patients who underwent open surgery than in those with a laparoscopic approach.

The risk of bile duct injury in relation to the surgical approach was calculated. In the EC group, we found 9 cases of bile duct injury in open surgery (6.8%) and 6 cases in the laparoscopic approach (1.52%), (*p* < 0.05). In the DC group, there was a higher tendency towards bile duct injury in open surgery (7.58%) than in laparoscopic surgery (3.22%), but the differences were not statistically significant.

### 3.10. Operating Time

There were no significant differences in operating time between the EC and DC groups (Table 6). The only factor that influenced the surgical time was the severity of the cholecystitis, with a significant increase in the time for moderate and severe cholecystitis cases (*p* < 0.05).

### 3.11. Reoperations

There were no significant differences between the percentage of reoperations in the EC group and the DC group (Table 6). In the multivariate analysis, it was noted that the EC group had almost twice the risk of reoperation of the DC group (OR = 1.90), but this ratio was not statistically significant (*p* > 0.05). It also highlighted that severe cholecystitis had a greater risk of reoperation (OR = 2.72), but it was not statistically significant (*p* > 0.05). The model has a low capacity to predict the risk of reoperation, with area under the ROC curve of 0.66.

### 3.12. Estimated Direct Cost Savings

Faced with the impossibility of assessing, using the data collected in the present study, the exact difference in costs between the treatment options of EC and DC, we instead made an approximation of the same, taking into account the three most important parameters with statistically significant differences between the two study groups: Emergency Department care for readmission, days of stay in ICU, and days of hospital stay.

The EC group had a total of 36 readmissions (0.068 readmissions per patient). The DC group had a total of 112 readmissions (0.219 readmissions per patient). If we subtract both figures we get 0.151, which is the difference in readmissions per patient between EC and DC.

The EC group had a total of 79 days of stay in the ICU (0.149 days of stay in ICU per patient). The DC group had a total of 372 days of stay in ICU (0.727 days of stay in ICU per patient). If we subtract both figures we get 0.578, which is the difference in days of stay in ICU per patient between EC and DC.

The EC group had an average of 8.9 days of hospital stay per patient and the DC group an average of 15.8 days of hospital stay per patient. If we subtract both figures we get 6.9, which is the difference in days of hospital stay per patient between EC and DC.

Therefore, each patient treated with EC would save 0.151 readmissions, 0.578 days of stay in ICU, and 8.9 days of hospitalization.

## 4. Discussion

In this comprehensive retrospective study, we compared, from a clinical point of view and in relation to the two possible surgical option treatments in the management of acute cholecystitis, the result of the treatment protocols for acute cholecystitis of two nearby hospitals, with very similar profiles in terms of capacity and organizational structure. Both hospitals provide health care to populations with similar demographic characteristics. We have contrasted the reality of the daily practice in the treatment of acute cholecystitis, without a selection of patients according to age, comorbidity, the severity of the acute cholecystitis or the surgical approach. In this way, our study has also included patients who underwent open surgery that had a greater proportion of cases of moderate and severe cholecystitis, factors that most influence, according to our data, the appearance of complications and mortality. This fact means that studies, which only include laparoscopic surgery cases, may have questionable external validity, due to dismissal of cases that have potentially worse evolution.

Of note, in the DC group, patients were operated on after an average of 105 days, an interval which is longer than the ideal 6–8-week period. This was due to the waiting list of a public hospital. We do not think that this influenced the morbidity rate, since fibrosis tends to decrease after several months.

Data from our study in relation to morbidity indicate that DC had a higher rate of complications than EC, due to a higher proportion of minor surgical complications. Many of the previously published studies also showed higher rates of morbidity in patients with DC, but from analyzing the morbidity of some of the most influential studies, such as the meta-analysis of Papi et al. [19] and Gurusamy and Samraj [12], we note that there were no significant differences between both groups. We have observed in our study that the patients who benefit most from EC were those with a Charlson index > 3 (greater age and comorbidity); concordantly, the risk of complications is 50% higher in patients with Charlson index > 3 who underwent DC. This fact has also been highlighted in recent studies [29–32].

We found no significant differences in morbidity among DC patients who underwent surgery during the first 72 hours and those who underwent surgery after 72 hours from the onset of symptoms. This result contrasts with the work published by González-Rodríguez et al. [33], where the morbidity was twice as high in surgery after 72 hours as in surgery within the first 72 hours and with the publication of Banz et al. [15], which notes higher rates of complications as the time increases in the evolution of acute cholecystitis.

The risk of postoperative infection was twice as high in the DC group as in the EC cases, which contrasts with the results of the meta-analysis of Gurusamy and Samraj [12], which notes a higher proportion of infections that required percutaneous drainage in the EC group. With regard to postoperative bleeding, we noted a lesser tendency towards bleeding in DC than in EC, which concurs with the results previously obtained by Norrby et al. [34]. We found that the proportion of bile leakage and major injuries of the bile duct was almost double in DC compared to that in EC, but with no statistically significant differences, which is in line with the results published by Gurusamy et al. in their various meta-analyses [12, 14, 16]. In our experience, the laparoscopic approach seems to be safer than the open surgery in the EC group, but we must consider that the selection of the type of surgery was not randomized in any case, so the results are not conclusive. We must point out that we believe that the ATOM [35] classification is the most appropriate form of assessment of iatrogenic injury to the bile duct, but, given its recent publication and the retrospective nature of the present study, which does not permit us to know certain aspects for the correct characterization of some of the injuries, we have used the Strasberg classification.

In relation to the morbidity analysis, there is a factor that has not been measured, which we believe may be of great interest and, in some way, a crucial factor in supporting the EC as the most optimal treatment for acute cholecystitis. This factor is the assessment, within the DC group, of the medical complications arising during the medical treatment of cholecystitis, as well as the deterioration in health of patients occurring between the index episode and the elective cholecystectomy.

Despite the fact that the mortality rate was more than twice as high in the DC group as in the EC group, the differences were not significant. The majority of the previous studies present similar mortality rates for both groups, with percentages close to 1% [15, 19] or without registered mortality [12, 14, 16].

EC patients had a significantly lower average hospital stay than that of DC patients. All of the articles published to date offer significantly lower results of hospital stay in the EC group, with differences in days of stay ranging from 2 days in the population study of Banz et al. [15] to 10 days in the van der Linden and Sunzel [4] and Papi et al. [19] studies. In addition, many of the works published hospital stay results very close to those of our EC group; among others, Lai et al. showed 7.6 days [7], Papi et al. 10.6 days [19], and Gurusamy et al. 6.7 days [14]. The patients who underwent surgery after 72 hours from the onset of symptoms presented an average stay significantly higher than the patients operated on within the first 72 hours, similar to the DC group, something that was pointed out previously by other studies [6–10, 36–38]; this is explained by the greater average stay prior to the surgery of the patients who underwent surgery after 72 hours of the symptoms onset.

The difference between the percentages of readmissions of the EC and the DC groups is due to the readmissions of the DC group that occur between the first admission for acute cholecystitis and the admission to perform the cholecystectomy (18.2%), which is somewhat lower than the results provided by Lahtinen et al. [39] and Lau et al. [40], with percentages of readmission prior to surgery between 25% and 30%.

We found a greater tendency towards reoperations in the EC group with respect to the DC group, which contrasts with the data published by Banz et al. [15] in their population study, where they highlight a greater proportion of reoperations in DC (27.9%) than in EC (11.9%).

As illustrated in the present study, the worst results of DC as compared to EC are due, in great measure, to the cases operated on earlier than expected and the nonoperated patients, something that is avoided with the EC protocols.

The average hospital stay and the percentage of patients who required a readmission, as well as the percentage of patients who were admitted to the ICU, were all significantly higher in the DC group than in the EC group. All of these factors contribute to ensuring that, with a high probability, the direct costs of EC treatment are lower than those of DC, something also pointed out by other recent studies [17, 41–43].

*Main Limitations of the Study*. (1) It is retrospective study. (2) Patients are treated in two different hospitals. That could induce variability in the surgical management or perioperative treatment. However, this variability would be minimal, since both hospitals are of the highest standard and both universities. (3) The study just analyzes two different treatment protocols; it is not an “intention to treat analysis” actually. 

*Conclusion*. EC provides better morbidity results, as well as a clear trend toward lower mortality and fewer injuries to the main bile duct. No differences were found in the rate of complications between patients who underwent surgery within the first 72 hours of symptoms and the patients operated on more than 72 hours after the initiation of symptoms. In addition, EC could be of benefit for elderly patients with high comorbidity and lead to a reduction in direct costs due to fewer stays in ICU, fewer readmissions, and fewer days of hospital stay. We would recommend DC only in cases where acute pancreatitis, choledocholithiasis, or cholangitis cannot be ruled out and those with unacceptable anesthetic risk at the time of diagnosis.

## Figures and Tables

**Table 1:** Comparison of the groups.

	EC (*n* = 531)	DC (*n* = 512)
Age	66.8	70.0
*Female* ^*∗*^, *n* (%)	326 (42.6)	206 (40.2)
*ASA* ^*∗*^, *n* (%)		
I	115 (21.6)	120 (23.4)
II	259 (48.8)	242 (47.3)
III	139 (26.2)	129 (25.2)
IV	18 (3.4)	21 (4.1)
Charlson	3.7	4.1
*Diabetics* ^*∗*^, *n* (%)	113 (22.1)	87 (17.0)
*Severity of cholecystitis* ^*∗*^, *n* (%)		
Mild	229 (43.1)	234 (45.7)
Moderate	262 (49.4)	235 (46.1)
Severe	40 (7.5)	42 (8.2)

^**∗**^ *p* > 0.05.

**Table 2:** Overall and surgical morbidity after surgery; mortality.

	Overall morb.	Surgical morb.	Mortality
*EC*	29.9%** (158)**	17.6%** (93)**	0.8% (6)
<72 h	29.6% (140)	16.9% (80)	0.4% (2)
>72 h	33.3% (18)	24.1% (13)	0%
No surgery			100% (4)
*DC*	38.7%** (157)**	29.9%** (123)**	2.7% (14)
Elective	22.4% (60)	18.3% (49)	0.4% (1)
Urgent	69.2% (97)	51.7% (74)	2.8% (4)
No surgery			8.9% (9)

Highlighted in bold font are statistically significant values.

**Table 3:** Overall complications according to the Clavien-Dindo scale.

	I-II	III-IV
*EC*	20.4%** (108)**	9.3% (49)
<72 h	20.1% (95)	9.3% (44)
>72 h	24.1% (13)	9.3% (5)
*DC*	29.7%** (122)**	8.3% (34)
Elective	19.0% (51)	3.0% (8)
Urgent	49.7% (71)	18.2% (26)

Highlighted in bold font are statistically significant values.

**Table 4:** Surgical complications according to the Clavien-Dindo scale.

	I-II	III-IV
*EC*	10.3%** (54)**	7.2% (37)
<72 h	9.5% (45)	7.2% (34)
>72 h	16.7% (9)	7.4% (4)
*DC*	23.1%** (95)**	6.6% (27)
Elective	15.3% (41)	3.0% (8)
Urgent	37.8% (54)	13.3% (19)

Highlighted in bold font are statistically significant values.

**Table 5:** Most relevant surgical complications.

	Infection	Bleeding	Bile injury	Strasberg D-E
*EC*	9.7%** (51)**	3.0% (16)	2.8% (15)	0.6% (3)
<72 h	9.3% (44)	2.5% (12)	2.7% (13)	0.6% (3)
>72 h	12.9% (7)	7.4% (4)	3.7% (2)	0%
*DC*	16.5%** (68)**	1.2% (5)	4.6% (19)	1.0% (4)
Elect.	9.3% (25)	0.7% (2)	2.2% (6)	0.8% (2)
Urg.	30.1% (43)	2.1% (3)	9.1% (13)	1.4% (2)

Highlighted in bold font are statistically significant values.

**Table 6:** Other results: days of hospital stay, readmissions, ICU stay, type of surgery, operating time, and reoperations.

	Hosp. stay	Readmiss.	ICU	LPS/CONV	Oper. time	Reoperat.
*EC*	**8.9 (ds 7.6)**	6.8%** (36)**	2.3%** (12)**	74.8%/21.6%	90.8 (ds 34.1)	4.6% (24)
<72 h	**8.1 (ds 7.6)**		2.1% (10)	75.1%/20.6%	91.1 (ds 38.6)	4.4% (21)
>72 h	**15.5 (ds 7.8)**		3.7% (2)	72.2%/30.8%	93.5 (ds 25.2)	5.6% (3)
*DC*	**15.8 (ds 12.2)**	21.9%** (112)**	7.8%** (32)**	67.9%/9.3%	89.5 (ds 28.1)	2.4% (10)
Elect.	13.6 (ds 11.9)		0%	91.4%/6.1%	84.2 (ds 18.9)	1.1% (3)
Urg.	19.9 (ds 12.2)		21.7% (31)	23.8%/32.3%	97.0 (ds 22.9)	4.9% (7)
No Surg.	15.8 (ds 12.2)		1% (1)			

Highlighted in bold font are statistically significant values.

## Additional Points

This paper is based on the thesis realized by Miguel Sánchez-Carrasco “Evaluation of Early Cholecystectomy versus Delayed Cholecystectomy in the Treatment of Acute Cholecystitis,” Department of Surgical Sciences, University of Cantabria, 2014.

## References

[1] Mulholland J. H., Ellison E. H., Friesen S. R. (1957). *Delayed Operative Management of Acute Cholecystitis. Current Surgical Management*.

[2] Ellison E. H., Miholland J. H., Friesen S. R. (1957). *Early Operation for Acute Cholecystitis. Current Surgical Management*.

[3] Pines B., Rabinowitch J. (1959). Perforation of the gallbladder in acute cholecystitis. *Annals of Surgery*.

[4] van der Linden W., Sunzel H. (1970). Early versus delayed operation for acute cholecystitis. A controlled clinical trial. *The American Journal of Surgery*.

[5] Kiviluoto T., Sirén J., Luukkonen P., Kivilaakso E. (1998). Randomised trial of laparoscopic versus open cholecystectomy for acute and gangrenous cholecystitis. *The Lancet*.

[6] Lo C.-M., Liu C.-L., Fan S.-T., Lai E. C. S., Wong J. (1998). Prospective randomized study of early versus delayed laparoscopic cholecystectomy for acute cholecystitis. *Annals of Surgery*.

[7] Lai P. B. S., Kwong K. H., Leung K. L. (1998). Randomized trial of early versus delayed laparoscopic cholecystectomy for acute cholecystitis. *British Journal of Surgery*.

[8] Chandler C. F., Lane J. S., Ferguson P., Thompson J. E., Ashley S. W. (2000). Prospective evaluation of early versus delayed laparoscopic cholecystectomy for treatment of acute cholecystitis. *The American Surgeon*.

[9] Serralta A. S., Bueno J. L., Planells M. R., Rodero D. R. (2003). Prospective evaluation of emergency versus delayed laparoscopic cholecystectomy for early cholecystitis. *Surgical Laparoscopy, Endoscopy and Percutaneous Techniques*.

[10] Johansson M., Thune A., Blomqvist A., Nelvin L., Lundell L. (2003). Management of acute cholecystitis in the laparoscopic era: results of a prospective, randomized clinical trial. *Journal of Gastrointestinal Surgery*.

[11] Kolla S. B., Aggarwal S., Kumar A. (2004). Early vs delayed laparoscopic cholecystectomy for acute cholecystitis: a prospective randomized trial. *Surgical Endoscopy and Other Interventional Techniques*.

[12] Gurusamy K. S., Samraj K. (2006). Early versus delayed laparoscopic cholecystectomy for acute cholecystitis (review). *Cochrane Database of Systematic Reviews*.

[13] Siddiqui T., MacDonald A., Chong P. S., Jenkins J. T. (2008). Early versus delayed laparoscopic cholecystectomy for acute cholecystitis: a meta-analysis of randomized clinical trials. *The American Journal of Surgery*.

[14] Gurusamy K. S., Samraj K., Gluud C., Wilson E., Davidson B. R. (2010). Meta-analysis of randomized controlled trials on the safety and effectiveness of early versus delayed laparoscopic cholecystectomy for acute cholecystitis. *British Journal of Surgery*.

[15] Banz V., Gsponer T., Candinas D., Güller U. (2011). Population-based analysis of 4113 patients with acute cholecystitis: defining the optimal time-point for laparoscopic cholecystectomy. *Annals of Surgery*.

[16] Gurusamy K. S., Davidson C., Gluud C., Davidson B. R. (2013). Early versus delayed laparoscopic cholecystectomy for people with acute cholecystitis. *The Cochrane Database of Systematic Reviews*.

[17] Gutt C. N., Encke J., Köninger J. (2013). Acute cholecystitis: early versus delayed cholecystectomy, a multicenter randomized trial (ACDc Study, nct00447304). *Annals of Surgery*.

[18] Vetrhus M., Søreide O., Nesvik I., Søndenaa K. (2003). Acute cholecystitis: delayed surgery or observation. A randomized clinical trial. *Scandinavian Journal of Gastroenterology*.

[19] Papi C., Catarci M., D'Ambrosio L. (2004). Timing of cholecystectomy for acute calculous cholecystitis: a meta-analysis. *The American Journal of Gastroenterology*.

[20] Senapati P. S. P., Bhattarcharya D., Harinath G., Ammori B. J. (2003). A survey of the timing and approach to the surgical management of cholelithiasis in patients with acute biliary pancreatitis and acute cholecystitis in the UK. *Annals of the Royal College of Surgeons of England*.

[21] Livingston E. H., Rege R. V. (2004). A nationwide study of conversion from laparoscopic to open cholecystectomy. *The American Journal of Surgery*.

[22] Feliu X., Targarona E. M., García A. (2003). La cirugía laparoscópica en España. Resultados de la encuesta nacional de la Sección de Cirugía Endoscópica de la Asociación Española de Cirujanos. *Cirugía Española*.

[23] Shikata S., Noguchi Y., Fukui T. (2005). Early versus delayed cholecystectomy for acute cholecystitis: a meta-analysis of randomized controlled trials. *Surgery Today*.

[24] Takada T., Strasberg S. M., Solomkin J. S. (2013). TG13: updated Tokyo Guidelines for the management of acute cholangitis and cholecystitis. *Journal of Hepato-Biliary-Pancreatic Sciences*.

[25] Strasberg S. M., Hertl M., Soper N. J. (1995). An analysis of the problem of biliary injury during laparoscopic cholecystectomy. *Journal of the American College of Surgeons*.

[26] Dindo D., Demartines N., Clavien P.-A. (2004). Classification of surgical complications. A new proposal with evaluation in a cohort of 6336 patients and results of a survey. *Annals of Surgery*.

[27] Dripps R. D., Lamont A., Eckenhoff J. E. (1961). The role of anesthesia in surgical mortality. *The Journal of the American Medical Association*.

[28] Charlson M. E., Pompei P., Ales K. L., MacKenzie C. R. (1987). A new method of classifying prognostic comorbidity in longitudinal studies: development and validation. *Journal of Chronic Diseases*.

[29] Rodríguez-Sanjuán J. C., Arruabarrena A., Sánchez-Moreno L., González-Sánchez F., Herrera L. A., Gómez-Fleitas M. (2012). Acute cholecystitis in high surgical risk patients: percutaneous cholecystostomy or emergency cholecystectomy?. *American Journal of Surgery*.

[30] Cheng Y., Leng J., Tan J., Chen K., Dong J. (2013). Proper surgical technique approved for early laparoscopic cholecystectomy for non-critically ill elderly patients with acute cholecystitis. *Hepato-Gastroenterology*.

[31] Ferrarese A. G., Solej M., Enrico S. (2013). Elective and emergency laparoscopic cholecystectomy in the elderly: our experience. *BMC Surgery*.

[32] Haltmeier T., Benjamin E., Inaba K., Lam L., Demetriades D. (2015). Early versus delayed same-admission laparoscopic cholecystectomy for acute cholecystitis in elderly patients with comorbidities. *Journal of Trauma and Acute Care Surgery*.

[33] González-Rodríguez F. J., Paredes-Cotoré J. P., Pontón C. (2009). Early or delayed laparoscopic cholecystectomy in acute cholecystitis? Conclusions of a controlled trial. *Hepato-Gastroenterology*.

[34] Norrby S., Herlin P., Holmin T., Sjödahl R., Tagesson C. (1983). Early or delayed cholecystectomy in acute cholecystitis? A clinical trial. *British Journal of Surgery*.

[35] Fingerhut A., Dziri C., Garden O. J. (2013). ATOM, the all-inclusive, nominal EAES classification of bile duct injuries during cholecystectomy. *Surgical Endoscopy*.

[36] Rattner D. W., Ferguson C., Warshaw A. L. (1993). Factors associated with successful laparoscopic cholecystectomy for acute cholecystitis. *Annals of Surgery*.

[37] Eldar S., Sabo E., Nash E., Abrahamson J., Matter I. (1997). Laparoscopic cholecystectomy for acute cholecystitis: prospective trial. *World Journal of Surgery*.

[38] Garber S. M., Korman J., Cosgrove J. M., Cohen J. R. (1997). Early laparoscopic cholecystectomy for acute cholecystitis. *Surgical Endoscopy*.

[39] Lahtinen J., Alhava E. M., Aukee S. (1978). Acute cholecystitis treated by early and delayed surgery. A controlled clinical trial. *Scandinavian Journal of Gastroenterology*.

[40] Lau H., Lo C. Y., Patil N. G., Yuen W. K. (2006). Early versus delayed-interval laparoscopic cholecystectomy for acute cholecystitis. *Surgical Endoscopy*.

[41] Macafee D. A. L., Humes D. J., Bouliotis G., Beckingham I. J., Whynes D. K., Lobo D. N. (2009). Prospective randomized trial using cost-utility analysis of early versus delayed laparoscopic cholecystectomy for acute gallbladder disease. *British Journal of Surgery*.

[42] Wilson E., Gurusamy K., Gluud C., Davidson B. R. (2010). Cost-utility and value-of-information analysis of early versus delayed laparoscopic cholecystectomy for acute cholecystitis. *British Journal of Surgery*.

[43] Johner A., Raymakers A., Wiseman S. M. (2013). Cost utility of early versus delayed laparoscopic cholecystectomy for acute cholecystitis. *Surgical Endoscopy and Other Interventional Techniques*.

